# Altered brain metabolism contributes to executive function deficits in school-aged children born very preterm

**DOI:** 10.1038/s41390-020-1024-1

**Published:** 2020-06-26

**Authors:** Barbara Schnider, Ruth Tuura, Vera Disselhoff, Bea Latal, Flavia Maria Wehrle, Cornelia Franziska Hagmann, the EpoKids Research Group

**Affiliations:** 1 Department of Neonatology and Pediatric Intensive Care, University Children’s Hospital Zurich, Zurich, Switzerland; 2 Children’s Research Center, University Children’s Hospital Zurich, Zurich, Switzerland; 3 Centre for MR Research, University Children’s Hospital Zurich, Zurich, Switzerland; 4 Child Development Center, University Children’s Hospital Zurich, Zurich, Switzerland

## Abstract

**Background:**

Executive function deficits in children born very preterm (VPT) have been linked to anatomical abnormalities in white matter and subcortical brain structures. This study aimed to investigate how altered brain metabolism contributes to these deficits in VPT children at school-age.

**Methods:**

Fifty-four VPT participants aged 8–13 years and 62 term-born peers were assessed with an executive function test battery. Brain metabolites were obtained in the frontal white matter and the basal ganglia/thalami, using proton magnetic resonance spectroscopy (MRS). *N*-acetylaspartate (NAA)/creatine (Cr), choline (Cho)/Cr, glutamate + glutamine (Glx)/Cr, and myo-Inositol (mI)/Cr were compared between groups and associations with executive functions were explored using linear regression.

**Results:**

In the frontal white matter, VPT showed lower Glx/Cr (mean difference: −5.91%, 95% CI [−10.50, −1.32]), higher Cho/Cr (7.39%, 95%-CI [2.68, 12.10]), and higher mI/Cr (5.41%, 95%-CI [0.18, 10.64]) while there were no differences in the basal ganglia/thalami. Lower executive functions were associated with lower frontal Glx/Cr ratios in both groups (*β* = 0.16, *p* = 0.05) and higher mI/Cr ratios in the VPT group only (interaction: *β* = −0.17, *p* = 0.02).

**Conclusion:**

Long-term brain metabolite alterations in the frontal white matter may be related to executive function deficits in VPT children at school-age.

**Impact:**

- Very preterm birth is associated with long-term brain metabolite alterations in the frontal white matter.
- Such alterations may contribute to deficits in executive function abilities.
- Injury processes in the brain can persist for years after the initial insult.
- Our findings provide new insights beyond structural and functional imaging, which help to elucidate the processes involved in abnormal brain development following preterm birth.
- Ultimately, this may lead to earlier identification of children at risk for developing deficits and more effective interventions.

## Introduction

Very preterm birth is a risk factor for long-term neurodevelopmental sequelae.^1,2^ Among the abilities most frequently impaired are executive functions,^3,4^ a set of higher-order and inter-related cognitive skills, responsible for adaptive and goal-directed behavior.^5,6^ Executive function deficits in children and adolescents born preterm have been linked to persisting structural brain alterations evident on MRI, such as white matter abnormalities and smaller volumes in subcortical structures including the caudate and thalamus.^7–10^ In parallel, altered neural activation patterns in executive function networks in response to tasks have been reported.^11–14^

Proton magnetic resonance spectroscopy (MRS) provides information beyond structural and functional imaging, enabling the in vivo estimation of brain metabolism by detecting different molecules in tissue noninvasively. Commonly investigated brain metabolites include *N*-acetylaspartate (NAA), choline-containing compounds (Cho), combined glutamate + glutamine (Glx), creatine (Cr), and lactate (Lac). Each metabolite is involved in multiple aspects of brain metabolism and characterizes different aspects of health and pathology.^15^

Previous studies revealed abnormal brain metabolism in preterm infants to be associated with white matter injury^16–20^ and reduced thalamic volume.^21^ Importantly, such alterations were related to poorer neurodevelopmental and motor outcomes in the first 2 years of life.^22–26^ In preterm children and adolescents, findings on brain metabolism are inconclusive. While there is some evidence suggesting similar metabolite levels in very preterm children and term-born peers,^27,28^ other studies observed metabolic alterations^29–31^ also linked to cognitive dysfunction.^32^ For a more comprehensive understanding on brain metabolism and potential associations with cognitive functions beyond infancy, more studies are needed.

Consequently, we aimed to investigate the impact of preterm birth on brain metabolism in school-aged children, in frontal white matter and deep gray matter structures—two regions particularly vulnerable in the preterm brain^33^—and, in addition, whether brain metabolism in those regions relates to executive function abilities, one of the most frequent deficits in contemporary cohorts of preterm children. We hypothesized that in our sample of children born very preterm, alterations in brain metabolism are observed in both regions and that these alterations contribute to deficits in executive function abilities.

## Material and methods

### Participants

This article reports on participants originally enrolled in the study entitled “Does erythropoietin improve outcome in very preterm infants?” (NCT00413946). Briefly, 450 infants born between 26 weeks and 31 weeks 6 days of gestation took part in a randomized, double-blind, placebo-controlled, multicenter trial in Switzerland between 2005 and 2012. Recruitment and follow-up of the initial trial to age 2 years have been described previously.^34–36^ The long-term follow-up study (EpoKids) examines whether early administration of high-dose erythropoietin (Epo) in very preterm infants has beneficial effects on executive functions, processing speed and global brain connectivity at school-age.^37^ Children were eligible for the EpoKids study if they were included in the 2-year follow-up (*n* = 365) of the initial trial.^35^ To date, children born between 2005 and 2009 have been approached for the Epokids study (*n* = 180). Of those, 100 (56%) were recruited successfully: Fourteen families agreed to complete the questionnaires only and seven children from French-speaking areas of Switzerland participated in a short assessment at the Geneva University Hospitals & University of Geneva. Those children were not further considered for the current analyses. This eventually results in a group of 79 participants born very preterm, aged 8−13 years at the time of assessment (see Supplementary Figure for a participant flow chart). Perinatal and routine follow-up data of participants born very preterm were obtained from the database of the initial trial. Comparisons between participants and nonparticipants revealed no group differences with regard to gestational age, birthweight, perinatal complications, motor and intellectual abilities, and head circumferences at 2 years of age (all *p* > 0.05). Socioeconomic status (SES) of participating families was higher than of nonparticipating families (mean = 7.4, SD = 2.1 and mean = 6.4, SD = 2.5, respectively; *p* = 0.004). Additionally, a control group of 78 term-born participants born between 2005 and 2009 was recruited. Inclusion criteria for controls comprised birth at term (i.e., ≥37 weeks; 0 days of gestation), no neonatal complications and no neurodevelopmental or neurologic illness past or present (i.e., attention-deficit/hyperactivity disorder, autism spectrum disorder, epilepsy, encephalopathy), as reported by the parents.

### Procedure

Study participants were examined at the University Children’s Hospital Zurich, either on weekdays or weekends, at the families’ convenience. The neurodevelopmental assessment was divided into two parts (one in the morning and the other in the afternoon), each lasting approximately 2 hours. To avoid systematic effects of fatigue, tasks were administered in a randomized order. Neurodevelopmental testing was succeeded by an MRI and MRS examination. All data were collected between July 2017 and September 2019. The study was approved by the local ethical committee (KEK 2017-00521). Participants were asked for their verbal consent and parents or a legal guardian provided written consent. All participating children were compensated with a gift voucher.

### Instruments and measures

#### Neurodevelopmental assessment

The neurodevelopmental tasks reported here have been described previously.^37^ In short, IQ was estimated with an abbreviated version of the Wechsler Intelligence Scale for Children (WISC-IV, German version).^38^ To assess processing speed, the symbol search and coding subtests of the WISC-IV^38^ were applied. Key aspects of executive function abilities (i.e. inhibition and interference, working memory, cognitive flexibility, fluency, and planning) were captured with standardized tasks.

To measure *inhibition abilities*, two tasks were applied. The stop-signal paradigm^39^ is a computerized task that requires participants to respond as quickly and accurately as possible to a go-stimulus (cartoon airplane) and to inhibit the response if a stop-stimulus (red frame around airplane) is presented. The dependent variable was the stop-signal reaction time, defined as the mean reaction time of the participant minus the mean delay of the stop-stimulus. The participants’ interference control was investigated by the Color-Word-Interference task of the Delis−Kaplan Executive Function System (D-KEFS).^40^ The participant is presented with color names written in an incongruent ink color and asked to name the ink color and ignore the written word. This requires the participant to inhibit the more automatic word reading response (i.e., the “Stroop” effect^41^). Completion time was used as dependent variable. *Working memory* was assessed by three tasks. The working memory subtest of the German “Test Battery for Attention Testing” (Testbatterie zur Aufmerksamkeitsprüfung; TAP)^42^ is a computerized two-back task. Participants are asked to indicate by button press if a briefly presented digit corresponds to the penultimately presented digit. The sum of omission and commission errors was used as dependent variable. In addition, the maximum backward span of the Corsi Block Tapping-Test,^43^ a visual-spatial working memory task, was used. Participants are instructed to tap a sequence of blocks in reverse order as shown by the examiner. Similarly, the maximum backward span of the subtest digit span (WISC-IV, German version)^38^ requires participants to repeat digit sequences in the reverse order in which they were read out. This served as dependent variable for verbal working memory. *Cognitive flexibility* was measured by two tasks. The Trail Making Task (D-KEFS)^40^ is a visuomotor sequencing task, which requires participants to switch between connecting numbers and letters in sequence. The completion time served as dependent variable. In the flexibility subtest of the TAP,^42^ participants are instructed to react alternately to changing target stimuli (i.e. round and angular shapes), which are presented in a pairwise manner. The number of errors served as dependent variable. *Fluency* was measured by two tasks. The Design Fluency task (D-KEFS)^40^ comprises three conditions: filled dots, empty dots, and switching. In each condition, the participants are instructed to draw different designs for 1 min using only four straight lines to connect the dots. The number of correct designs was used as the dependent variable. Additionally, participants’ verbal fluency was assessed by three subtests of the Regensburger Wortflüssigkeitstest (RWT),^44^ a German-language verbal fluency test that requires participants to produce words in accordance with specific rules: In the semantic fluency subtest, types of animals have to be generated. In the switching subtests, words starting with the letters “G” and “R” (phonetic switching) and types of fruits and sports (semantic switching), respectively, have to be named in an alternating manner. The total number of correctly produced words in 2 minutes was used as dependent variables. *Planning and problem-solving abilities* were assessed with the Tower subtest of the D-KEFS,^40^ in which participants have to displace disks from a starting to an ending position by following a set of rules. A total achievement score, derived from the sum of total points achieved for all items, was recorded and served as a dependent variable (i.e. correct towers within the time limit with the fewest moves achieving the highest number of points). Reasons for the missing data included technical problems, measurement errors, time constraints, or fatigue effects. Parental SES (ranging from 2 to 12) was estimated based on information on maternal and paternal education.

#### Magnetic resonance spectroscopy

**Fig. 1:**
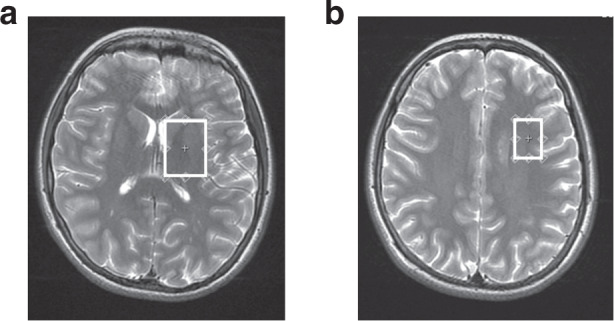
Location of voxels of interest (VOIs). VOIs were positioned in the **a** left basal ganglia to include the head of the caudate, putamen and internal capsule (20 × 25 × 35 mm^3^) and **b** left dorsolateral frontal white matter (20 × 16 × 24 mm^3^).

**Fig. 2:**
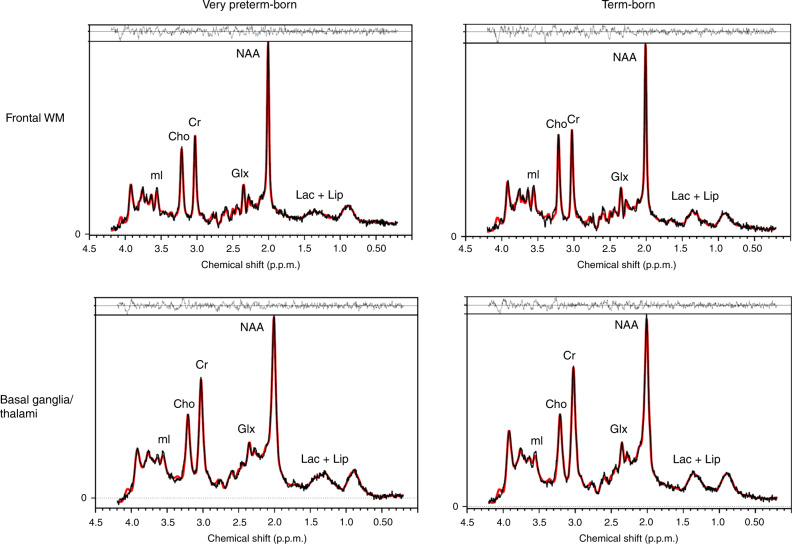
Examples of representative spectra. Representative spectra from the dorsolateral frontal white matter (top) and basal ganglia/thalami (bottom) from a very preterm participant (left) and a term-born participant (right). The spectral data are depicted in black with the LCModel fit overlaid in red. The residuals between the data and the fit are shown above each spectrum.

MRS was performed on a 3 T GE MR750 scanner (GE Healthcare, Milwaukee, WI, USA) using an eight-channel receive-only head coil. Proton (^1^H) MRS was acquired from two voxels placed in the basal ganglia/thalami (20 × 25 × 35 mm^3^) and dorsolateral frontal white matter (20 × 16 × 24 mm^3^), respectively (Fig. 1), using a single voxel Point Resolved (PRESS) acquisition (repetition time = 3000 ms, echo time = 35 ms, 96 averages). Spectra were quantified using LCModel.^45^ The LCModel analyzes in vivo spectra as a linear combination of modeled in vitro spectra from individual metabolites. With this method, metabolite concentrations and their uncertainties (Cramer−Rao lower bounds), expressed as percentages of the amplitude (SD), are obtained^46^ (Fig. 2). The MRS signals analyzed in this study included NAA, Cho (i.e., glycerol-phosphocholine (GPC) and phosphocholine (PCh)), Glx, myo-inositol (mI), Cr (i.e., combined creatine and phosphocreatine) and Lac. Metabolite ratios to Cr were calculated. Each spectrum was visually inspected for the presence of artifacts or fitting errors. If SD > 10% for NAA or Cr, respectively, the spectra was completely excluded, and if Cho, Glx or mI had an SD > 20%, individual metabolites were excluded. Because of overall low Lac levels in the healthy brain and to obtain a generally more representative data sample in this cohort, no SD cut-off was applied with regard to Lac.

The comprehensive scan protocol included volumetric, structural, and functional imaging. For the localization of the spectroscopy voxels in each participant, T1-weighted MR images with a 3D fast spoiled gradient echo sequence (echo time = 5 ms, repetition time = 11 ms, inversion time = 600 ms, flip angle = 8°, field of view (FOV) = 25.6 cm, reconstruction matrix: 256 × 256) were applied. Magnetic resonance spectroscopy data were missing because of refusal or fatigue (frontal white matter *n* = 23, basal ganglia/thalamus: *n* = 27), poor data quality (frontal white matter: *n* = 14, basal ganglia/thalamus: *n* = 16, e.g. due to braces), time constraints (*n* = 2) or technical reasons (*n* = 2) (see Supplementary Figure for a participant flow chart). To test for a potential selection bias, participants born very preterm with and without MRS data were compared with regard to perinatal, socio-demographic, and cognitive data. These analyses revealed similar characteristics in both groups (all *p* > 0.05).

### Statistical analyses

Raw scores of each executive function task were *z*-transformed using the mean and standard deviation of the term-born group. This provided equally scaled results for each of the ten tasks. A mean *z*-score was then calculated for each of the five assessed domains of executive functioning if the data for all the corresponding tasks were available. The domain-specific *z*-scores were subsequently averaged to a global score if at least four out of the five domain-specific scores were available. The global executive function score served as an overall estimate of the participants’ executive function abilities. Similar approaches have been used previously to estimate global executive function abilities in children born very preterm.^47,48^

Descriptive statistics included mean and standard deviation for the continuous variables and numbers and percentages of total for the categorical variables. Demographic and neurodevelopmental data were compared between groups using independent samples *t* test, Wilcoxon rank-sum test, or chi-squared test, as appropriate.

The relationship between brain metabolite ratios and global executive function abilities was explored through linear regression models with a separate model for each metabolite ratio—executive function global score combination. As covariates, SES, sex, age at assessment, and processing speed were included. An interaction term of birth status and MRS ratio was added, to test if there are differential relationships with outcome between the two groups. Only significant interaction terms remained in the models. In addition, to account for missing data in the covariates SES and processing speed, respectively, multivariate imputation by chained equations (MICE)^49^ was applied (see Supplementary Tables).

Normality of residuals was checked by visual inspection of histograms and QQ plots for raw data and residual analysis for linear models, respectively. Ninety-five percent confidence intervals (CI) are reported where relevant. Two-sided *p* values < 0.05 were considered significant. To account for multiple comparisons, *p* values were adjusted according to the Benjamini−Hochberg procedure (Tables 2 and 3).^50,51^ All statistical analyses were performed using R statistical software, Version 3.5.1.^49,52–59^

To ensure continued blinding in the EpoKids study, the initial allocation to treatment (i.e. Epo vs. placebo) was encoded by the study staff of the original trial (i.e. as intervention 1 vs. intervention 2). To address potential group differences between the treatment groups, neurodevelopmental outcome and MRS data were compared using independent samples *t* test. No group differences were detected. Consequently, the allocation to treatment group was not considered for further analyses and data of preterm participants were pooled.

## Results

Demographic and neurodevelopmental data on participants with a global executive function score are shown in Table 1. No group differences between very preterm and term-born participants emerged for sex and age at assessment. Socioeconomic status was higher in families of term-born participants. The processing speed index and the estimated IQ were lower in the very preterm group compared to the term-born controls.

**Table 1:** Sample characteristics of very preterm and term-born participants.

	VPT (*n* = 68)	TB (*n* = 70)	*p*
Female	29 (42.7)	34 (48.6)	0.60
Age at assessment (in years), mean (SD), range	10.8 (1.2), 8.8–13.4	11.1 (1.3), 8.2–13.5	0.23
Socioeconomic status^a^, mean (SD), range	7.9 (2.0), 4–12	9.6 (2.0), 6–12	<0.001
Gestational age (in weeks), mean (SD), range	29.3 (1.7), 26.0–31.7	39.7 (1.1), 37.3–42.0	<0.001
Birthweight (in g), mean (SD), range	1209 (320), 570–2020	3484 (466), 2370–4410	<0.001
Major brain lesions^b^	2 (2.9)	–	
BPD^b,c^	8 (11.8)	–	
NEC^b^	3 (4.4)	–	
ROP^b^ ≥ grade 3	0 (0.0)	–	
IQ estimate^d^, mean (SD), range	102.8 (15.3), 67.1–138.0	115.6 (13.7), 83.7–152.0	<0.001
Processing speed index^e^, mean (SD), range	100.9 (10.6), 76.0–129.0	106.9 (11.5), 83.0–131.0	0.002

Data are *n* (%) unless specified.

*VPT* very preterm participants, *TB* term-born participants, *BPD* bronchopulmonary dysplasia, *NEC* necrotizing enterocolitis, *ROP* retinopathy of prematurity.

^a^Information on families’ socioeconomic status was missing for five VPT and four TB participants.

^b^Perinatal data of term-born children as reported by parents. Inclusion criteria for TB children comprise no perinatal complications.

^c^Defined as requirement for additional oxygen at 36 0/7 weeks postmenstrual age.

^d^IQ estimate was missing for six VPT and one TB participants.

^e^Processing speed index was missing for five VPT and two TB participants.

### Global executive function score

**Fig. 3:**
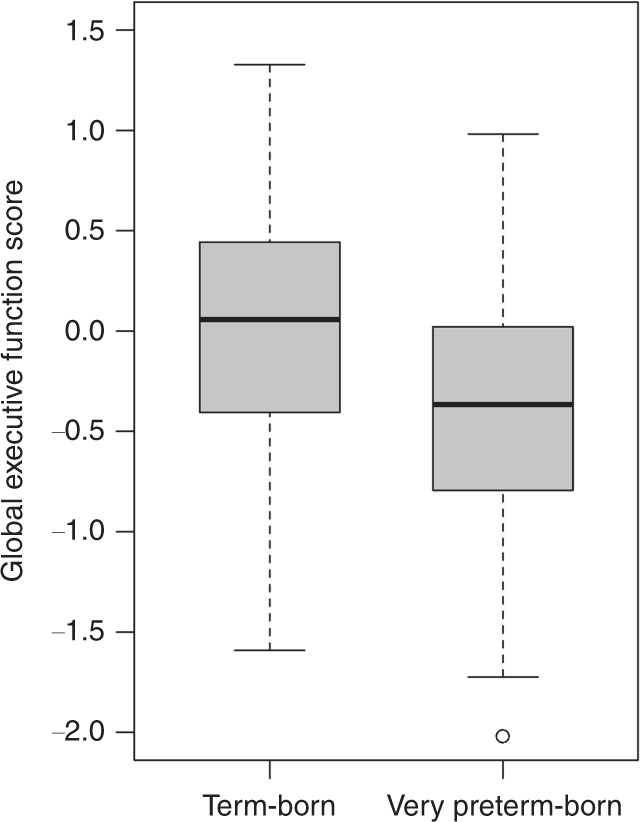
Global executive function *z*-scores in both birth groups. The upper whisker is either the maximum score or the third quartile plus 1.5 times the interquartile range (the length of the box). The lower whisker is either the minimum score or the first quartile minus 1.5 times the interquartile range.

Table 2 summarizes the results of the executive function assessment. The global executive function score in the very preterm group was 0.7 standardized mean differences (SMD; defined as the difference between the group means divided by the pooled SD) lower than in term-born group (Fig. 3). This difference between birth groups persisted, when controlling for age at assessment, sex, SES, and processing speed (*F*(5, 123) = 32.69, adjusted *R*^2^ = 0.55, *p* < 0.001; *B* = −0.21, 95% CI [−0.38, −0.05], *β* = −0.17, *p* = 0.01). In this model, age at assessment (*B* = 0.23, 95% CI [0.17, 0.30], *β* = 0.47, *p* < 0.001) and processing speed (*B* = 0.02, 95% CI [0.01, 0.02], *β* = 0.32, *p* < 0.001) were also associated with the global executive function score. For a detailed description of missing values in independent variables and the estimated model after performing multiple imputation, see Supplementary Tables 1 and 2.

**Table 2:** *Z*-scores of individual executive function tasks, aggregated domains, and the global executive function score in very preterm and term-born participants.

**Global score**^**a**^	**Domain**^**b**^					**Task**		
		VPT/TB *n*	VPT mean (SD)	TB mean (SD)	VPT vs TB *p*		VPT/TB *n*	VPT^c^ mean (SD)
*n* VPT/TB: 68/70 VPT/TB mean (SD) −0.39 (0.60)/0.04 (0.57) VPT vs TB *p* ≤ 0.001	Inhibition	68/69	−0.45 (0.86)	−0.01 (0.80)	0.004	Stop-signal, *reaction time*	69/70	−0.62 (1.14)
						D-KEFS CWIT, *Inhibition completion time*	78/74	−0.30 (1.03)
	Working memory	76/74	−0.56 (0.93)	0.02 (0.77)	<0.001	WISC-IV digit span, *maximum span backward*	77/88	−0.22 (0.86)
						Corsi Block, *maximum span backward*	79/77	−0.72 (1.12)
						TAP Working Memory, *Errors*	77/75	−0.82 (1.73)
	Cognitive flexibility	68/73	−0.35 (0.88)	0.03 (0.78)	0.009	D-KEFS TMT, *Switching completion time*	68/76	−0.64 (1.33)
						TAP Flexibility, *Errors*	77/75	−0.14 (1.05)
	Fluency	70/75	−0.44 (0.52)	0.01 (0.71)	<0.001	D-KEFS Design Fluency^d^, *correct designs: Condition 1–3*	77/76	−0.48 (0.62)
						RWT Verbal Fluency^e^, *correct words: G/R-words, Animals, Sport/fruit*	72/77	−0.41 (0.74)
	Planning	66/55	−0.22 (1.17)	0.01 (1.0)	0.25	D-KEFS Tower, *Total achievement score*	66/55	−0.22 (1.17)

*VPT* very preterm participants, *TB* term-born participants.

To account for multiple comparisons, *p* values were adjusted according to the Benjamini−Hochberg procedure.

^a^The global score was calculated if at least four out of the five domain-specific scores were available (80%).

^b^A domain-specific score was calculated if the data for all the corresponding tasks were available.

^c^*z*-scores of TB participants had mean = 0 and SD = 1 on task level.

^d^For the design fluency task, a *z*-score was calculated for each of the three conditions separately. Subsequently, a mean *z*-score was calculated if at least two of the three conditions were complete.

^e^The verbal fluency *z*-score was calculated identically as the design fluency score.

### MRS metabolite ratios

Magnetic resonance spectroscopy data are summarized in Table 3. Compared to term-born participants, very preterm participants had higher Cho/Cr, higher mI/Cr, and lower Glx/Cr ratios in the frontal white matter, with all except mI/Cr surviving Benjamini–Hochberg correction for multiple comparisons. Frontal NAA/Cr did not differ between participants born very preterm and term-born peers. Lac/Cr was not reliably detected in our cohort and is therefore not reported here. No group differences in MRS metabolite ratios were apparent in the basal ganglia/thalamus voxel.

**Table 3:** MRS metabolite ratios in very preterm and term-born participants.

Metabolite ratio	VPT (*n* = 54)	TB (*n* = 62)	Mean difference (95% CI)		*p*^b^	*p*^c^
	Mean (SD)	Mean (SD)	Absolute difference	% difference^a^		
Frontal WM						
NAA/Cr	1.64 (0.13)	1.65 (0.14)	−0.01 (−0.06, 0.05)	−0.33 (−3.39, 2.72)	0.83	0.83
Cho/Cr	0.32 (0.04)	0.29 (0.04)	0.02 (0.01, 0.04)	7.39 (2.68, 12.10)	0.003	0.01
Glx/Cr^d^	1.63 (0.19)	1.73 (0.25)	−0.10 (−0.02, −0.18)	−5.91 (−10.50, −1.32)	0.01	0.03
mI/Cr	0.55 (0.07)	0.52 (0.08)	0.03 (0.00,0.06)	5.41 (0.18, 10.64)	0.04	0.06
Basal ganglia/thalami	(*n* = 49)	(*n* = 61)				
NAA/Cr	1.35 (0.13)	1.34 (0.12)	0.02 (−0.03, 0.06)	1.25 (−2.29, 4.79)	0.49	0.92
Cho/Cr	0.23 (0.03)	0.23 (0.03)	0.00 (−0.01, 0.01)	−1.13 (−5.23, 2.97)	0.59	0.92
Glx/Cr^d^	1.42 (0.27)	1.43 (0.25)	0.00 (−0.10, 0.09)	−0.33 (−7.17, 6.51)	0.92	0.92
mI/Cr	0.34 (0.09)	0.34 (0.10)	0.00 (−0.04, 0.03)	−0.52 (−11.00, 9.96)	0.92	0.92

*VPT* very preterm participants, *TB* term-born participants, *CI* confidence interval, *WM* white matter.

^a^Metabolite ratio difference expressed as a percentage of the mean ratio in the controls for the particular metabolite ratio.

^b^Unadjusted *p* values.

^c^To account for multiple comparisons, *p* values were adjusted according to the Benjamini−Hochberg procedure.

^d^Two participants (one VPT, one TB) had to be excluded for the analysis of Glx/Cr ratios due to artifacts in the peaks.

### Relationship between MRS metabolite ratios and the global executive function score

**Fig. 4:**
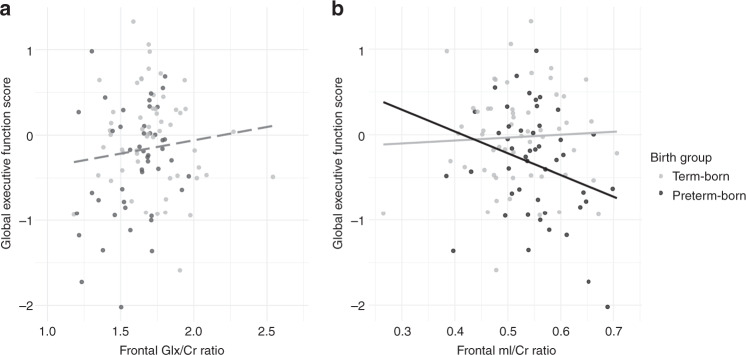
Associations between the global executive function score and frontal Glx/Cr ratios (Fig. 4a) and mI/Cr ratios (Fig. 4b). **a** The dashed regression line represents the unadjusted association between frontal Glx/Cr ratios and the executive function global score. Since there was no significant interaction effect in the adjusted regression model (Table 4, Model 1), one regression line was calculated for both groups. Gray dots represent term-born participants, black dots very preterm participants. **b** The two regression lines represent the unadjusted relation between frontal mI/Cr ratios and the executive function global score in both groups separately, since the birth group × frontal mI/Cr interaction term was significant in the adjusted regression model (Table 4, Model 2). Grey dots represent term-born participants, black dots very preterm participants.

Relationships between MRS metabolite ratios and the global executive function score were explored for frontal Cho/Cr, Glx/Cr, and mI/Cr, since significant differences between birth groups were apparent (unadjusted *p* values). Frontal Cho/Cr ratios were not significantly associated with the global executive function score (*B* = −0.01, 95% CI [−2.56, −2.54], *β* = −0.001, *p* = 0.99). Frontal Glx/Cr and frontal mI/Cr ratios were significantly related to the global executive function score: Table 4 shows the results from the regression analysis separately for frontal Glx/Cr (Model 1) and mI/Cr (Model 2) as independent variables. Higher Glx/Cr ratios in the frontal white matter were associated with a better global executive function score in both groups (Fig. 4a) when adjusting for age at assessment, sex, SES, and processing speed. When adding this metabolite ratio to the model, the effect of birth group diminished to nonsignificant (Table 4, Model 1). Increased mI/Cr ratios were related to lower global executive function score when adjusting for the same covariates. This association was observed in the very preterm group, but not in the term-born group (*p* value for interaction = 0.02) (Table 4, Model 2*;* Fig. 4b). See Supplementary Table 3 and Supplementary Table 4 for a detailed description of missing values and the estimated model after performing multiple imputation.

**Table 4:** Multiple linear regression models to explore the relationship between the global executive function score and frontal Glx/Cr (Model 1) and mI/Cr ratios (Model 2).

	Outcome: Global executive function score									
	Model 1: Estimated model including Glx/Cr					Model 2: Estimated model including mI/Cr				
	(*F*(6, 89) = 18.61, *p* < 0.001), adjusted *R*^*2*^ = 0.53					(*F*(7, 90) = 17.16, *p* < 0.001), adjusted *R*^*2*^ = 0.54				
	*B*	*SE B*	95% CI	*β*	*p*	*B*	*SE B*	95% CI	*β*	*p*
Intercept	−6.01	0.74	−7.48, −4.53		<0.001	−4.46	0.60	−5.65, −3.27		<0.001
Age at assessment	0.23	0.04	0.15, 0.31	0.46	<0.001	0.22	0.04	0.15, 0.30	0.48	<0.001
Female sex	0.12	0.09	−0.06, 0.31	0.10	0.18	0.15	0.09	−0.08, 0.29	0.12	0.11
SES	0.05	0.02	0.01, 0.10	0.18	0.03	0.05	0.02	−0.00, 0.10	0.17	0.04
Processing speed	0.02	0.00	0.01, 0.03	0.37	<0.001	0.02	0.00	0.01, 0.03	0.33	<0.001
Preterm birth	−0.06	0.11	−0.27, 0.16	−0.04	0.59	–	–	–	–	–
Glx/Cr^a^	0.44	0.22	0.00, 0.87	0.16	0.05					
mI/Cr × birth group^b^						−2.84	1.21	−5.25, −0.44	−0.17	0.02

*SES* socioeconomic status, *B* unstandardized regression coefficients, *SE* *B* standard error of *B*, *CI* confidence interval, *β* standardized regression coefficients.

^a^The interaction effect is omitted from the model since it was nonsignificant.

^b^Main effects are not presented since they cannot be interpreted in case of a significant interaction effect.

## Discussion

We aimed to compare brain metabolism and explore the association with executive functioning in a cohort of children born very preterm and their term-born peers at school-age.

In very preterm children, we found higher Cho/Cr, lower Glx/Cr, and a trend towards higher mI/Cr ratios in the left frontal white matter. In parallel, we confirmed global executive function deficits in these children. Further, frontal Glx/Cr and mI/Cr ratios were related to executive function abilities, suggesting long-term brain metabolite alterations partly explaining executive function deficits in children born very preterm.

Our results on lower frontal Glx/Cr ratios in children born very preterm are in line with the literature from preterm survivors at term-equivalent age: Kwon et al.^60^ found lower right frontal GABA and glutamate concentrations in preterm infants compared to a term-born control group. Also, lower Glx levels in a white matter voxel (i.e. in the centrum semiovale) have been reported previously in preterm neonates.^19^ For early childhood, previous studies of preterm cohorts contrast our results: At age 3–4 years, Glx/Cr ratios in the left frontal periventricular white matter were found to be higher in children with very low-birth-weight (VLBW) compared to controls, although the group difference was driven by decreased Cr levels in the VLBW group rather than increased Glx.^30^ At 4 and 6 years of age, no differences in frontal Glx concentrations were found between children born very preterm and term-born controls.^27^ Glutamate is mainly stored in neurons and acts as a precursor for GABA in neurons and glutamine in astrocytes.^61,62^ Together, glutamate and glutamine form an important neurotransmitter cycle, but in addition, their roles in the brain may be far more complex.^61,62^ During periods of brain development and maturation, glutamate plays an important role in different stages of neurogenesis including progenitor proliferation, migration, differentiation and survival, synaptogenesis and spinogenesis.^63,64^ Interestingly, in rat pups, exposure to maternal isolation and repetitive neonatal pain was found to produce an increase in serum corticosterone and decreased glutamate/Cr levels in the frontal cortex and in the hippocampus.^65^ Also, frontal Glx/Cr ratios were reduced in maternally isolated pups compared to nonisolated pups, altogether suggesting alterations in stress response and neurochemistry in reaction to early-life stressors.^65^ In the neonatal intensive care unit, preterm infants are exposed to various stressors including pain and reduced maternal care, both of which might impact further development.^66^ Whether these early adverse environmental factors similarly contribute to altered Glx levels at school-age warrants further investigation.

Our findings on Cho/Cr ratios are consistent with a previous study reporting increased Cho/Cr ratios in the frontal white matter in VLBW children in early childhood.^30^ In contrast, others suggest similar Cho/Cr ratios in frontal white matter in preterm and term-born children at 4 and 6 years of age^27^ and adolescents at 15 years of age.^31^ Further, Cheong et al.^32^ found decreased Cho/Cr ratios in a sample of extremely preterm adolescents, but in a different brain region (i.e. the left posterior cingulate white matter). The MRS signal from Cho is thought to represent the structural integrity and signaling of cell membranes and to reflect unbound Cho-containing molecules that are components of the myelin sheath.^15,67^ Developmentally, Cho decreases in the first year of life and remains relatively stable across childhood.^68^ The pathophysiological importance of elevated Cho has been attributed to demyelination, membrane breakdown, and inflammation;^69^ thus, an increase in Cho signaling has been reported in white matter pathologies such as traumatic brain injury, where elevated Cho levels distinguished children with traumatic brain injury from controls even in normal-appearing white matter.^70^ Additionally, in this group of patients, a persistent activation of microglia as a sign of a chronic inflammatory response has been shown several years after the insult occurred.^71^ Therefore, the observed alterations in the Cho resonance in our cohort of preterm children may reflect variations in membrane turnover (i.e. either increased membrane synthesis or breakdown) or changes in cell density,^15^ particularly since inflammation is a contributing factor in the pathophysiology of encephalopathy of prematurity.^33^ Hence, based on the present findings and the literature, we can only speculate that membrane disturbances causing altered Cho levels are a result of cerebral white matter injury following preterm birth.

The higher mI/Cr ratios in children born very preterm compared to term-born peers in this cohort remained trend-level only after correction for multiple comparison. Since mI is only detectable with short echo-times, there are fewer MRS studies investigating mI levels compared to other metabolites in preterm individuals. In preterm neonates, one study reported higher levels of mI/Cr ratios in neonates with white matter damage compared to neonates with normal-appearing white matter.^17^ Further, preterm infants with diffuse white matter injury visible on MR imaging showed no age-related decline on mI/Cr levels, whereas in infants without such MR signal abnormalities, the expected age-associated decrease of mI has been reported.^72^ In early childhood, however, no alterations were found in white matter mI levels following preterm birth.^27,30^ The importance of mI lies in its role as a regulator of brain osmotic balance and through its involvement in complex cellular processes during typical development, including cell migration.^15,73^ Previous MRS studies support the idea that an increase in mI reflects astrocyte proliferation.^69,74^ Abundant reactive astrocytes and activated microglia have been discussed to be involved in cerebral white matter injury in preterm infants.^75–77^ Thus, potentially, the increased mI/Cr ratio observed in our cohort reflects cellular vulnerability and developmental disruption involving astrocytes and microglia attributed to preterm birth. Alterations in the above-mentioned metabolites may also reflect the tertiary phase after injury to the immature brain years after the initial insult.^77^

In our analysis, no significant effect of preterm birth was observed for frontal NAA/Cr ratios. Reports from studies investigating NAA in children and adolescents born preterm range from no apparent differences to term-born controls^27,28,31^ to lower NAA levels in preterm individuals.^29,30,32^ The utility of NAA as a universal neuronal marker has been questioned, probably depending on the underlying pathology. In addition, an involvement of NAA in oligodendrocyte myelin formation and intracellular signaling has been discussed (for an overview, see ^15^). Importantly, a recovery of NAA levels has been reported after insult.^78^ Against this background, our findings may either indicate a normalization of NAA levels after preterm birth or reflect metabolic changes without involving aberrant NAA metabolism at this developmental stage.

To date, there are no studies reporting on Lac levels in childhood and adolescence following preterm birth. Lac is only present in very low concentrations in healthy tissue in the brain, and thus not generally observable in conventional MR spectra.^15^ Accordingly, we did not reliably detect Lac/Cr ratios in our cohort of preterm individuals with low rates of brain lesions. In full-term neonates with hypoxic-ischemic brain injury, deep grey matter Lac/NAA is a well-established biomarker for poor neurodevelopmental outcome.^79–81^ Interestingly, in preterm infants, increased Lac levels have been reported in the context of acute white matter injury at term-equivalent age and were related to illness severity.^16,18^ However, the role of Lac for later life remains unclear in the very preterm population.

To our knowledge, our analysis is the first to report Glx/Cr in a subcortical voxel in children born very preterm at school-age. No evidence emerged for Glx/Cr alterations in children born very preterm in subcortical brain structures. For NAA, Cho, and mI, we also did not observe group differences within the subcortical voxel. This is in line with a previous study in younger children born very preterm, reporting no alterations in brain metabolism in a basal ganglia voxel.^28^ More studies are needed to confirm that brain metabolism in subcortical brain structures is not affected in very preterm children. This might also help to shed light on how differences between studies regarding perinatal characteristics, age at assessment, variability regarding brain regions of interest, and MRS methods applied contribute to some of the ambiguous findings on brain metabolism following preterm birth.

There is little information on potential associations between brain metabolism and functional outcomes in the preterm population beyond the first years of life: In early childhood, no associations between brain metabolism in a basal ganglia voxel and cognitive outcomes were found.^28^ In contrast, altered frontal white matter NAA/Cho, Glx/Cr, and Cho/Cr co-occurred with lower levels of early executive functioning in preschoolers born preterm.^30^ Recently, a study with adolescents born extremely preterm revealed lower NAA/Cr and Cho/Cr ratios in the posterior cingulate white matter to be associated with poorer cognitive functioning.^32^ In fact, a considerable number of studies report on long-term adverse effects of very preterm birth on white matter microstructure throughout childhood and adolescence^82,83^ related to executive function abilities using diffusion tensor imaging.^9,84–87^

The present analysis contributes to this area of research by focusing on higher-order cognitive abilities, since in today’s cohorts of very preterm-born children at school-age such deficits are among the most frequent.^1,4,88,89^ In line with the literature, we report lower executive function performance in very preterm participants. Importantly, lower frontal Glx/Cr ratios were related to poorer executive function abilities regardless of birth status (i.e. born very preterm or born at term). This result may indicate that aberrant Glx signaling is involved in deficits in higher cognitive processing. In fact, glutamate and glutamine have previously been found to be involved in metabolic processes related to cognitive functioning: In healthy young adults, in vivo prefrontal cortex glutamate levels were elevated during a working memory task (i.e. one of the core executive function abilities), reflecting increased metabolic activity and excitatory neurotransmission as a result of working memory-related demands.^90^ In adults with attention-deficit/hyperactivity disorder—a condition strongly linked to executive function deficits^91^—reduced Glx/Cr ratios^92^ and abnormal glutamate signaling^93,94^ were reported. Also, in children with autism spectrum disorder, another clinical population with frequent executive function deficits, white matter Glx was reported to be lower.^95^

Interestingly, mI/Cr ratios were differentially associated with executive functioning in children born very preterm and term-born controls: In children born very preterm, higher mI/Cr ratios were related to lower executive function abilities whereas no association was found in term-born children. These results suggest that even subtle changes in mI metabolism in the white matter may have a negative impact on executive function abilities. Previously, higher mI white matter levels have been observed in children with developmental delay around 6–7 years of age, but this difference disappeared around 9–10 years of age.^95^ To date, there are no studies exploring the relationship between higher-order cognitive functions and mI levels in typically developing term-born children or in individuals born very preterm.

In the very preterm group, we found altered frontal Cho/Cr ratios, but a functional relationship to these altered Cho/Cr ratios was not observed. This is consistent with a finding on adolescents born extremely preterm: Cho/Cr ratios in the posterior cingulate were not associated with working memory and attention,^96^ although a positive correlation between Cho/Cr ratios and IQ was reported in the same sample.^32^ Since the very preterm group in our analysis comprises relatively healthy and high-functioning children, we focused on deficits in higher cognitive abilities and their relationship with brain metabolism. Indeed, executive function deficits have been reported previously in children and adolescents born very preterm with normal general intellectual abilities,^97^ and one study observed an increased rate of executive function deficits in very preterm children born in recent birth cohorts,^89^ highlighting the importance of comprehensively investigating mechanisms underpinning these deficits.

### Limitations

In this analysis, we report on cross-sectional data. Eventually, only longitudinal studies will shed light on developmental trajectories of brain metabolism, and therefore allow conclusions to be drawn about whether altered brain metabolism remains a risk factor for impaired neurodevelopment as children born very preterm grow up. Our sample consisted of children born very preterm with relatively high estimated general cognitive abilities, a rather high socioeconomic background, and few neonatal complications. Further, the comprehensive study protocol of the EpoKids study led to a smaller sample size when compared to the number of children eligible for the present analysis. Overall, this may limit the generalizability of the results for the general population of children born very preterm. However, the sample size is still large when compared to other studies in this research area.^27,29–31^

Since the recruitment of the EpoKids study is ongoing, the potential effect of Epo on brain metabolites and outcome was not assessed in the analyses. Comparisons within the very preterm group did not, however, reveal any differences between the two treatment groups. This is in line with the findings of Gasparovic et al.,^27^ reporting no significant differences in metabolite levels at 4 and 6 years of age between children born preterm treated with erythropoiesis-stimulating agents and those receiving placebo shortly after birth.

Further, some technical limitations should be considered. In this analysis, we regarded Cr as a reference to quantify brain metabolites. Cr levels remain relatively stable after the first year of life^68^ and are therefore often used as a reference in MRS ratios. However, Cr levels are also associated with the degree of vascularization and the amount of activity occurring in a particular region.^15^ Thus, whether differences between the two birth groups are due to changes in the metabolite of the denominator, the numerator or both remains unknown. However, since the significant findings in our analysis, including higher Cho/Cr and lower Glx/Cr ratios in very preterm participants, go in different directions, it is unlikely that they are driven by group differences in Cr.

Additionally, we did not distinguish between glutamate and glutamine since the two metabolites show overlapping resonance frequencies (chemical shifts) at 3 T. Shifts from glutamate to glutamine, or vice versa, are therefore not detectable in our analysis. Observations of lower Glx/Cr may reflect lower glutamate levels, a finding that awaits further investigation with newer acquisition techniques and higher field-strengths. Regardless of resolving the individual components of Glx, the measured glutamate cannot be distinguished between its role as a neurotransmitter and metabolic purposes.

## Conclusion

Results from this study suggest that very preterm birth is associated with long-term brain metabolite alterations in the frontal white matter, partly explaining deficits in executive function abilities. Further research will help to improve the understanding of brain−behavioral relationships in children born very preterm and may ultimately lead to earlier identification of children at risk for developing deficits and more effective interventions.

## Supplementary information

Supplementary Tables

Supplementary Figure
