# Supplementary material for: Altered brain metabolism contributes to executive function deficits in school-aged children born very preterm
Source: Pediatr Res. 2020 Jun 26;88(5):739–48. doi: 10.1038/s41390-020-1024-1 (PMC7577839; doi:10.1038/s41390-020-1024-1)
Supplement: Supplementary file 1 — Supplementary Tables [file 41390_2020_1024_MOESM1_ESM.docx]

Supplemental Tables.

Missing values description and approach

Supplementary Table S1. Missing data of independent variables in the model related to children with Global (n = 138)

|  | Missing values |
| --- | --- |
| Age | 0 |
| Female sex | 0 |
| SES | 7/138 (5.1%) |
| Processing speed | 2/138 (1.4%) |
| Preterm birth | 0 |

SES: socioeconomic status

As illustrated in Table 2 of the main manuscript, the model is based on 138 observations, given that SES was not available for 4 families, and 2 children had no data on processing speed. Multiple imputation was applied to complement the rate of missing values in independent variables.

Supplementary Table S2. Global executive function score. Estimated model after multiple imputation

| adjusted *R^2^* = 0.54, 95% CI [0.42, 0.64] | | | | |
| --- | --- | --- | --- | --- |
|  | *B* | *SE* *B* | 95% CI | *p* |
| Intercept | -4.85 | 0.45 | -5.73, -3.96 | <.001 |
| Age | 0.24 | 0.03 | 0.17, 0.30 | <.001 |
| Female sex | 0.05 | 0.07 | -0.10, 0.19 | .53 |
| SES | 0.04 | 0.02 | -0.00, 0.08 | .06 |
| Processing speed | 0.02 | 0.00 | 0.01, 0.02 | <.001 |
| Preterm birth | -0.20 | 0.08 | -0.36, -0.04 | .01 |
| SES: socioeconomic status  *B*: unstandardized regression coefficients, SE *B*: standard error of *B,* CI: confidence interval | | | | |

Supplementary Table S3. Missing data of independent variables in the model related to children with MRS data and EF global scores

|  | Missing values |
| --- | --- |
| Age | 0 |
| Female sex | 0 |
| SES | a) 4/102 (3.9%) / b) 4/104 (3.8%) |
| Processing speed | a) 2/102 (2.0%) / b) 2/102 (1.9%) |
| Preterm birth | 0 |

SES: socioeconomic status

As illustrated in Table 4 of the main manuscript, a) the model including Glx/Cr is based on 96 instead of 102 children, b) the model including mI/Cr is based on 98 instead of 104 children, respectively, given that SES was not available for 4 families, and 2 children had no data on processing speed. Multiple imputation was applied to complement the rate of missing values in independent variables.

Supplementary Table S4. Multiple linear Regression models after multiple Imputation to explore the relationship between the global executive function score and frontal Glx/Cr and mI/Cr ratios

|  | Outcome: Global executive function score | | | | | | | | |
| --- | --- | --- | --- | --- | --- | --- | --- | --- | --- |
|  | Model 1:  Estimated model including **Glx/Cr** | | | |  | Model 2:  Estimated model including **mI/Cr** | | | |
| adjusted *R^2^* = 0.54, 95%-CI [0.39, 0.67] adjusted *R^2^* = 0.55, 95%-CI [0.41, 0.67] | | | | | | | | | |
|  | *B* | *SE* *B* | 95% CI | *p* |  | *B* | *SE* *B* | 95% CI | *p* |
| Intercept | -6.09 | 0.70 | -7.49, -4.69 | <.001 |  | -4.51 | 0.58 | -5.66, -3.36 | <.001 |
| Age at assessment | 0.24 | 0.04 | 0.16, 0.31 | <.001 |  | 0.24 | 0.04 | 0.17, 0.31 | <.001 |
| Female sex | 0.13 | 0.09 | -0.05, 0.30 | .16 |  | 0.15 | 0.09 | -0.03, 0.32 | .09 |
| SES | 0.05 | 0.02 | 0.00, 0.10 | .03 |  | 0.05 | 0.02 | 0.00, 0.09 | .04 |
| Processing speed | 0.02 | 0.00 | 0.01, 0.03 | <.001 |  | 0.02 | 0.00 | 0.01, 0.03 | <.001 |
| Preterm birth | -0.04 | 0.10 | -0.24, 0.16 | .72 |  |  |  |  |  |
| **Glx/Cr^a^** | **0.46** | **0.21** | **0.04, 0.88** | **.03** |  |  |  |  |  |
| **mI/Cr * birth group^b^** |  |  |  |  |  | **-2.86** | **1.16** | **-5.15, -0.56** | **.02** |
| SES: socioeconomic status  *B*: unstandardized regression coefficients, SE *B*: standard error of *B,* CI: confidence interval  ^a^The interaction effect is omitted from the model since it was non-significant. ^b^Main effects are not presented since they cannot be interpreted in case of a significant interaction effect. | | | | | | | | | |
