# Supplementary figures and images for: Altered brain metabolism contributes to executive function deficits in school-aged children born very preterm
Source: Pediatr Res. 2020 Jun 26;88(5):739–48. doi: 10.1038/s41390-020-1024-1 (PMC7577839; doi:10.1038/s41390-020-1024-1)

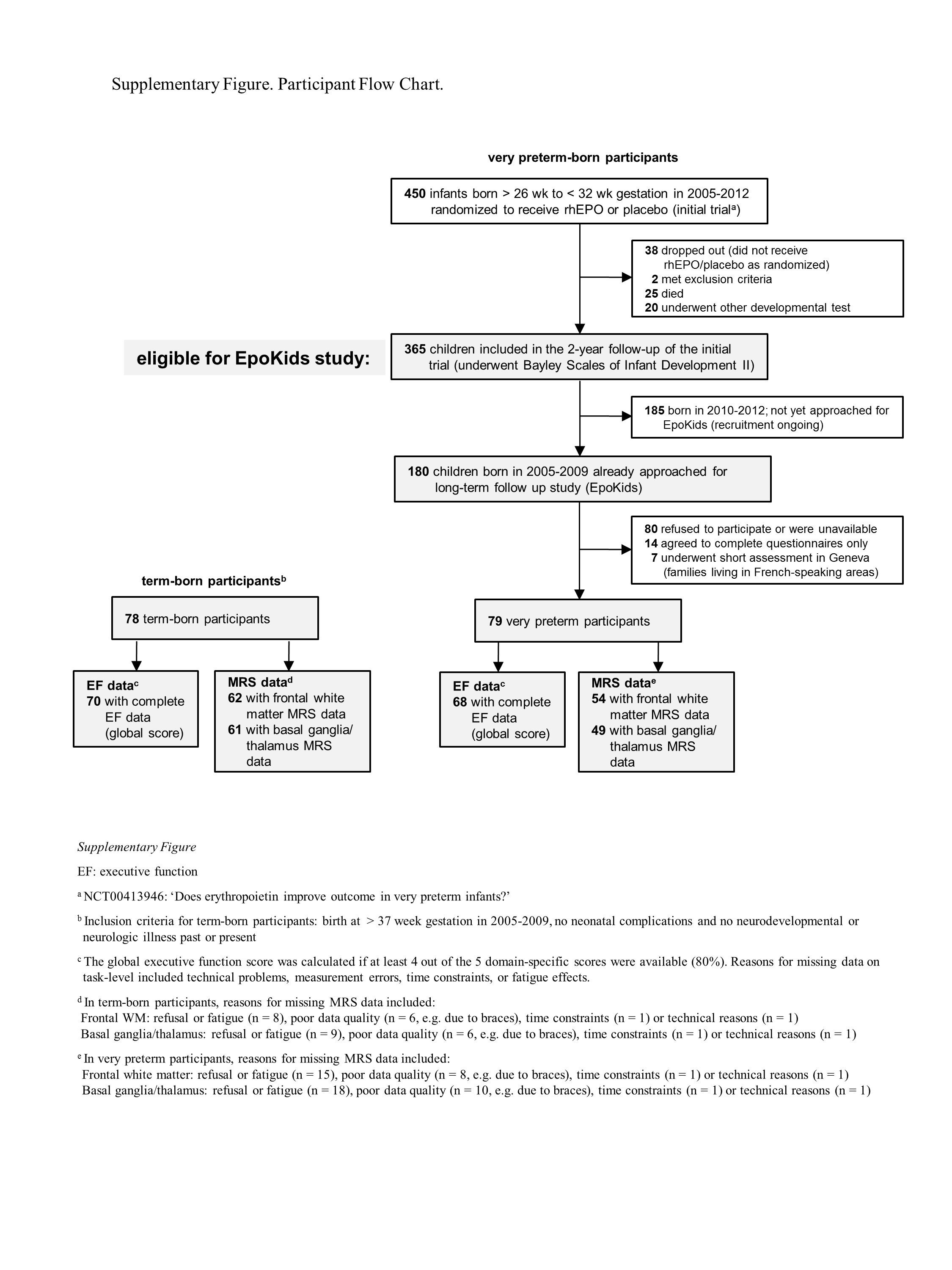

Supplement: Supplementary file 2 — Supplementary Figure [file 41390_2020_1024_MOESM2_ESM.jpg]
